# Boron-Implanted Silicon Substrates for Physical Adsorption of DNA Origami

**DOI:** 10.3390/ijms19092513

**Published:** 2018-08-24

**Authors:** Sadao Takabayashi, Shohei Kotani, Juan Flores-Estrada, Elijah Spears, Jennifer E. Padilla, Lizandra C. Godwin, Elton Graugnard, Wan Kuang, Scott Sills, William L. Hughes

**Affiliations:** 1 Micron School of Materials Science & Engineering, Boise State University, Boise, ID 83725, USA; sadaotakabayashi@u.boisestate.edu (S.T.); shoheikotani@u.boisestate.edu (S.K.); juanflores-estrada@u.boisestate.edu (J.F.-E.); elijahspears@u.boisestate.edu (E.S.); jenniferepadilla@boisestate.edu (J.E.P.); lizandragodwin@boisestate.edu (L.C.G.); eltongraugnard@boisestate.edu (E.G.); 2 Department of Electrical & Computer Engineering, Boise State University, Boise, ID 83725, USA; wankuang@boisestate.edu; 3 Micron Technology, Inc., 8000 South Federal Way, Boise, ID 83707-0006, USA; ssills@micron.com

**Keywords:** molecular self-assembly, DNA nanotechnology, DNA origami, electrostatics, semiconductor

## Abstract

DNA nanostructures routinely self-assemble with sub-10 nm feature sizes. This capability has created industry interest in using DNA as a lithographic mask, yet with few exceptions, solution-based deposition of DNA nanostructures has remained primarily academic to date. En route to controlled adsorption of DNA patterns onto manufactured substrates, deposition and placement of DNA origami has been demonstrated on chemically functionalized silicon substrates. While compelling, chemical functionalization adds fabrication complexity that limits mask efficiency and hence industry adoption. As an alternative, we developed an ion implantation process that tailors the surface potential of silicon substrates to facilitate adsorption of DNA nanostructures without the need for chemical functionalization. Industry standard 300 mm silicon wafers were processed, and we showed controlled adsorption of DNA origami onto boron-implanted silicon patterns; selective to a surrounding silicon oxide matrix. The hydrophilic substrate achieves very high surface selectivity by exploiting pH-dependent protonation of silanol-groups on silicon dioxide (SiO_2_), across a range of solution pH values and magnesium chloride (MgCl_2_) buffer concentrations.

## 1. Introduction

The semiconductor industry has followed Moore’s observation that the number of components per integrated circuit would increase exponentially with time [1,2]. This trend has been reinforced by decades of top-down scaling of photolithography. Today, 193 nm immersion lithography prints up to ~10^14^ features in a single exposure, at spatial pitches down to 80 nm [3]. Below the diffraction limit of light, 40 and 20 nm pitches are routine with self-aligned double patterning (SADP) [4] and quadruple patterning (SAQP) [5]. While extreme ultraviolet lithography is projected to extend direct-print lithography to a 32 nm pitch [6], SADP is still required to extend it below 20 nm [7]. In response to the escalating cost of photolithography [8,9,10], directed self-assembly (DSA) of block copolymers (BCP) have been explored [11,12,13,14,15,16]. While compelling, critical challenges have gated adoption of DSA-BCP technology as a volume manufacturing technology. First, its line edge roughness and critical dimension uniformity are too high when compared to traditional lithography. In addition, macro-molecular defects are very difficult to characterize in real-time during manufacturing. To overcome these challenges, programmable molecules such as DNA are starting to be explored as an alternative to BCP for patterning sub-lithographic features [17,18,19,20]. With a theoretical feature resolution of ~3 nm [21,22], and the ability to incorporate programmable optical defect metrology [23], DSA of DNA origami [24] or bricks [25] offers potential for sub-10 nm patterning [26,27,28].

Similar to block copolymers, DNA selectively adsorbs onto surfaces with favorable thermodynamic interactions. Adsorption is directed by pre-patterning a substrate with regions, or boundaries, that chemically differentiate favorable and unfavorable binding sites. For example, Sarveswaran et al. adsorbed DNA origami onto a positively charged self-assembled monolayer (SAM) that was pre-patterned on a silicon substrate [29]. Binding and non-binding sites were differentiated using aminopropyltrimethoxysilane (APTES) and a native oxide, respectively. In contrast, Gopinath et al. attached DNA origami onto a pre-patterned silicon dioxide substrate with negatively charged functional groups [30]. The binding and non-binding sites were differentiated using silanol and hexamethyldisilazane (HMDS) [30].

In general, surface differentiation via SAM layers is not desirable because it increases the complexity of the system and becomes a source of additional defects [31]. An alternative to SAM functionalization is to differentiate the surface using pre-patterned materials [32,33]. While DNA routinely adsorbs onto negatively charged mica surfaces [24,34], the binding strength of the DNA is non-uniform on the surface because of heterogeneous ionic exchange [35]. As an alternative to mica, Kershner et al. physically adsorbed DNA origami onto electron-beam patterned and topographically isolated diamond-like carbon sites [32]. Although their approach produced elevated surface densities, diamond-like carbon is sensitive to its processing conditions and not always amenable to DNA adsorption [30] Instead of growing a layer of material onto a substrate surface, altering the silicon surface property is a simpler approach to promote DNA origami adsorption.

To bring directed self-assembly of DNA closer to semiconductor manufacturing, we developed a boron-implantation process to tailor the surface potential of silicon substrates for physical adsorption of DNA origami. Physical adsorption of the origami was studied as a function of the deposition buffer pH, as well as the MgCl_2_ concentration. Origami adsorption was characterized as a function of pH on: (I) A boron-implanted silicon substrate, and (II) a thermally grown silicon dioxide (SiO_2_) substrate. Industry standard 300 mm wafers were also patterned in a semiconductor manufacturing facility with boron-implanted silicon features separated by a SiO_2_ matrix. Selective adsorption of DNA origami was then demonstrated on the implanted silicon surface with no deposition on SiO_2_.

## 2. Results

**Figure 1:**
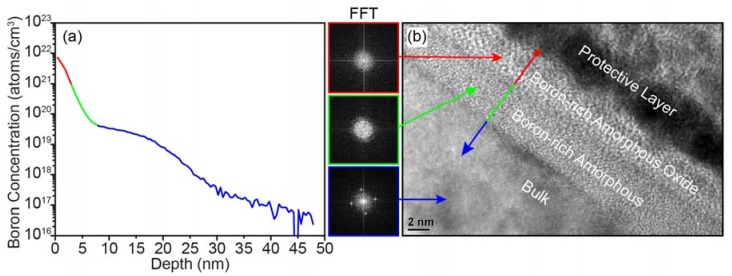
The Secondary Ion Mass Spectroscopy (SIMS) and cross-sectional Transmission Electron Microscopy (TEM) analyses of the boron-implanted silicon substrate revealed: (**a**) The boron concentration depth profile, and (**b**) structure of the substrate. The boron concentration at the surface of the substrate was ~14.6 atomic percent, and the majority of the boron was found within ~7 nm from the surface. The red, green, and blue colors on the plot (**a**) and the line in (**b**) correspond to the boron-rich amorphous oxide layer, the boron-rich amorphous layer, and the bulk silicon, respectively. The cross-sectional TEM image and the Fast-Fourier Transforms (FFTs) of the image showed that both outer layers were amorphous, and that the bulk was crystalline. The platinum protective layer was deposited onto the substrate surface prior to fabrication of the TEM specimen using focused-ion beam (FIB).

DNA origami triangles were modified from a previously reported study [24], as described in Supplement S1, and custom boron-implanted and SiO_2_ p-type silicon wafers were prepared by Micron Technology, as described in Supplement S2. The boron-implanted silicon substrate was characterized using X-ray Photoelectron Spectroscopy (XPS), Secondary Ion Mass Spectroscopy (SIMS), Transmission Electron Microscopy (TEM), and spectroscopic ellipsometry. According to XPS analysis of the as-received wafer, a 2–3 nm boron-rich oxide layer had formed within the substrate surface during the annealing process. As seen in Figure 1a, SIMS analysis of the as-received wafer showed that the boron concentration was ~14.6 atomic percent (at. %) at the surface, well beyond the <1 at. % solubility limit (~1 × 10^20^ per cm^3^) [36,37,38], and that the majority of the implanted boron was within ~7 nm of the surface. Figure 1b shows a cross-sectional TEM image of the as-received boron-implanted silicon substrate prepared by focused-ion beam (FIB) sectioning. The image reveals two layers above the bulk crystalline silicon, and the combined thickness of these layers agrees with the boron-rich region of the SIMS depth profile, which is colored to match the layers observed in the TEM image. Fast-Fourier Transforms (FFTs) of the layers in the TEM image revealed that the boron-rich surface layers were both amorphous. The outer layer appears inhomogeneous in the TEM cross-section images, possibly revealing boron precipitates within the boron-rich amorphous oxide [38,39]. Given the very high concentration of implanted boron, a boron-silicon phase is expected to have formed at the interface of the amorphous layer and the crystalline silicon [40], and this layer is known to be resistant to etching in hydrofluoric acid (HF), although the etch rates depend strongly on the etchant and boron concentration [40,41]. After cleaning the substrate with Piranha and 1:100 hydrofluoric acid (Piranha + HF, Supplement S1), the boron concentration at the substrate surface was determined to be ~2.5 at. %, which still exceeds the solubility limit of boron in silicon and represents the boron-rich amorphous layer. The resulting boron-rich layer was uniformly distributed over a ~1 cm × 1 cm area of the substrate surface, based on XPS. For characterization details, see Supplement S3. The boron-implantation greatly reduced the hydrophobicity of the substrate surface, as shown in Supplement S4. Similarly, a boron and phosphorus co-doped silicon surface is also hydrophilic and is known to possess negative potential [42]. Thus, we expect that the boron-implanted silicon substrate also possesses a surface potential.

**Figure 2:**
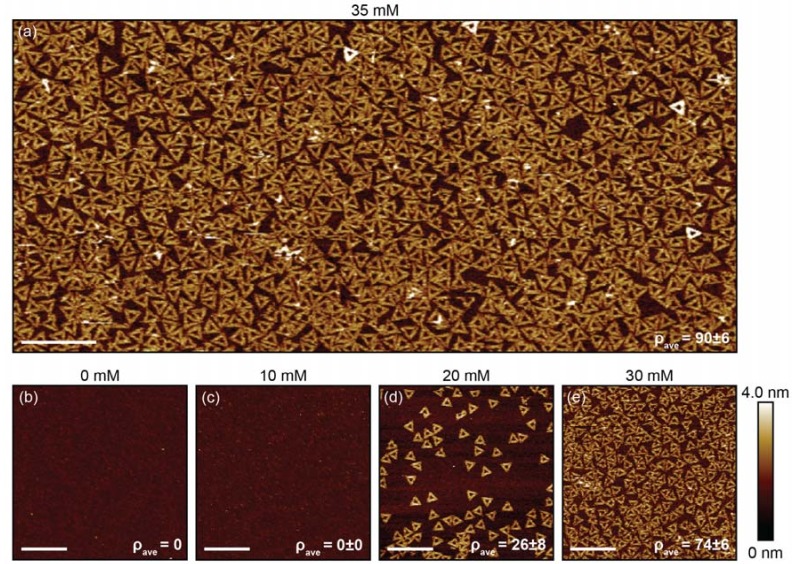
Atomic force microscopy (AFM) images of DNA origami triangles adsorbed onto boron-implanted silicon substrates. Prior to the DNA origami triangle deposition, the samples were cleaned with Piranha + hydrofluoric acid (HF). For all conditions, the deposition buffers were 10 mM bis-tris hydrochloric acid (HCl) with a pH of 6.6 and the deposition incubation time was ~24 h. The MgCl_2_ concentration ([MgCl_2_]) for (a) was 35 mM. The average surface density increased from 0 to 90 ± 6/μm^2^ as the [MgCl_2_] increased from 0 to 35 mM. Scale bars are 500 nm.

DNA origami triangles were deposited onto freshly cleaned boron-implanted silicon substrates with a deposition buffer using the procedure described in Supplement S1. As seen in Figure 2a, uniform, high density deposition of DNA origami triangles was observed. The average surface density (ρ_ave_) of DNA origami triangles was 90 ± 6/µm^2^. While the average surface density was statistically comparable to that of naturally occurring mica, the adsorption uniformity was greater (see Supplement S5). The DNA origami were incubated with the substrate for ~24 h since this incubation time was observed to give the highest surface density, as shown in Supplement S6. For longer deposition times, the density decreased by ~20%, likely due to changes in the surface chemical state. Since the time needed to reach monolayer DNA origami deposition depends on the origami concentration, shorter deposition times are expected for higher DNA origami concentrations during incubation. For the DNA nanostructure counting protocol, see Supplement S7.

The concentration of divalent magnesium ions is known to strongly affect the properties of DNA nanostructures, especially deposition onto substrates. DNA origami triangles were deposited onto boron-implanted silicon substrates to study how the MgCl_2_ concentration ([MgCl_2_]) in the deposition buffer impacted the average surface density. The [MgCl_2_] was varied from 0–35 mM and the deposition buffer pH was held constant at 6.6. As shown in Figure 2, DNA origami did not adhere at 0 mM and the average surface density increased from 0 to 90 ± 6 per µm^2^ as the [MgCl_2_] increased from 0 to 35 mM. Similar to deposition on mica, the deposition on the boron-implanted Si wafers shows a strong dependence on the [MgCl_2_].

**Figure 3:**
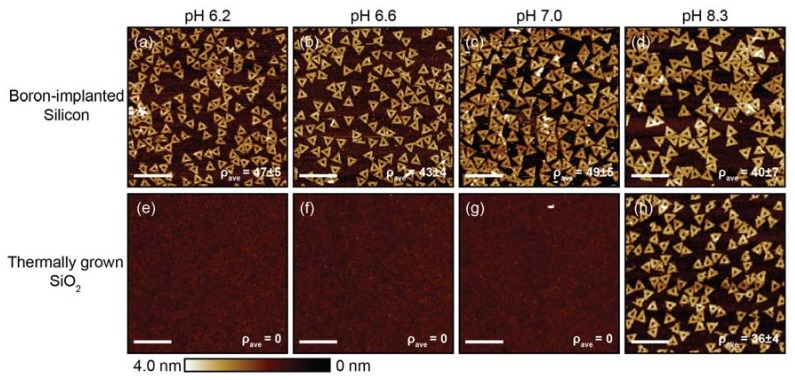
AFM images of DNA origami triangles adsorbed onto boron-implanted silicon substrates (**a**–**d**) and thermally grown silicon dioxide (SiO_2_) substrates (**e**–**h**) as a function of deposition buffer pH. The substrates were cleaned with Piranha + HF. For the pH values below 8.3, the deposition buffer was 10 mM bis-tris HCl with a [MgCl_2_] of 35 mM. For the pH value of 8.3, 1× tris-acetate- ethylenediaminetetraacetic acid (EDTA) with a [MgCl_2_] of 35 mM was selected to stay within the buffer range. For all conditions, the deposition incubation time was ~1 h. Scale bars are 500 nm.

Physical adsorption of DNA origami was studied for a fixed [MgCl_2_] of 35 mM in a deposition buffer pH range between 5.8 and 8.3 on the boron-implanted silicon substrate and a thermally grown SiO_2_ substrate (Figure 3). The average surface density range of 39 to 49 nanostructures per µm^2^ was observed on the boron-implanted silicon surface. In comparison, the surface density was significantly lower for the thermally grown SiO_2_ substrate, showing nearly zero adsorption at pH values between 5.8 and 7.2, and ~40 nanostructures per µm^2^ at a pH value of 8.3. For consistency, the thermally grown SiO_2_ substrate was also cleaned with Piranha + HF, which was not expected to completely remove the 100 nm thick oxide. The surface density seen in Figure 3b is lower compared to Figure 2a due to different incubation periods of ~1 h and ~24 h, respectively. For an expanded DNA origami adsorption dataset, see the atomic force microscopy (AFM) images in Supplement S8. The relationship between the DNA origami adsorption and the thickness of the boron-rich amorphous oxide on the boron-implanted silicon substrates is shown in Supplement S9. These data indicate that selective adsorption of DNA origami should be possible on a substrate with lithographically defined boron-implanted silicon features that are separated by a SiO_2_ matrix.

**Figure 4:**
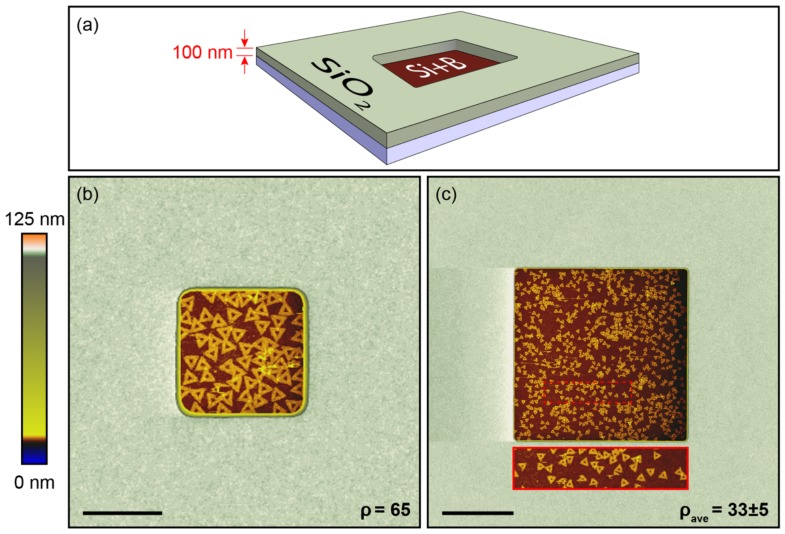
Schematic of a boron-implanted silicon feature surrounded by 100 nm thick SiO_2_ (**a**). AFM height images of lithographically fabricated 1 μm × 1 μm (**b**) and 5 μm × 5 μm (**c**) boron-implanted silicon features surrounded with SiO_2_. The z-axis color scale was adjusted to show nanometer-scale contrast both within the well and on SiO_2_ (100 nm above the well). The deposition buffer was 10 mM bis-tris HCl, with a pH of 6.6, and a [MgCl_2_] of 35 mM. DNA origami triangles adsorbed with a surface density of 65/μm^2^ in (**b**) and 33 ± 5/μm^2^ in (**c**) on the boron-implanted silicon surfaces, while no DNA origami adsorbed onto the SiO_2_ surfaces. The rectangular insert is a 2× magnified image of the boron-implanted silicon surface shown in (**c**). For both samples, the deposition incubation time was ~1 h. Scale bars are 600 nm for (**b**) and 2 μm for (**c**).

To test this hypothesis and demonstrate selective adsorption, DNA origami triangles were then deposited onto a patterned substrate with boron-implanted silicon features, including 1 µm × 1 µm and 5 µm × 5 µm in size wells, separated by a 100 nm thick, thermally grown SiO_2_ matrix. An optical image of the patterned wafer is shown in Supplement S2. The substrates were cleaned with Piranha + HF, and then DNA origami was deposited from a deposition buffer with an optimized pH of 6.6 and [MgCl_2_] of 35 mM. As shown in Figure 4, selective adsorption of DNA origami was achieved on boron-implanted silicon features but not on the SiO_2_ matrix. Unlike Gopinath et al. [30], spatial homogeneity was observed in the corners, edges, and interior of the patterned features, reflecting bulk adsorption from the solution, rather than 2D surface diffusion-limited coverage.

## 3. Discussion

**Figure 5:**
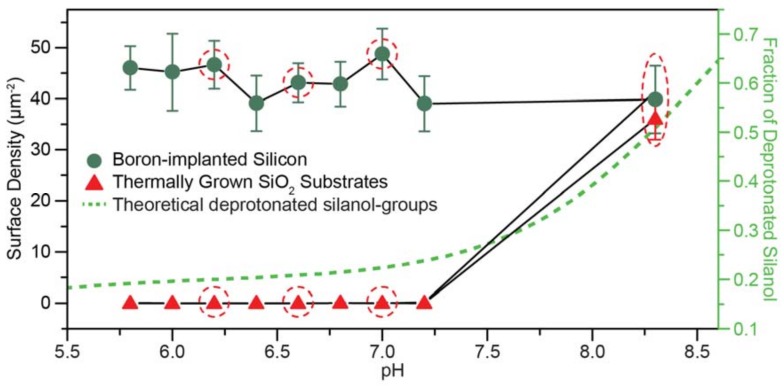
DNA origami surface density on boron-implanted silicon substrates oxide (green circle) and thermally grown SiO_2_ substrates (red triangle) as a function of deposition buffer pH. Representative data points for the AFM images in Figure 3 are highlighted with red circles, and the theoretical population fraction of deprotonated silanol-groups on the oxide surfaces is depicted with a green dotted line.

Physical adsorption of charged nanostructures in an electrolyte on silicon substrates is extremely complicated and includes pH-dependent specific ion effects on the structure and density of water and ions at the surface [43,44]. However, all pH values studied here are above the isoelectric point for SiO_2_, and the results are consistent with behavior described by Derjaguin, Landau, Verwey, and Overbeek (DLVO) theory [45,46], where deposition, or the lack thereof, is due to a net electrostatic attraction or repulsion between the substrate and the DNA origami nanostructures. Briefly, in the DNA origami buffer solution, an electric double layer forms around both the DNA origami, and at the boron-silicon surface. The thicknesses of both double layers depend on the electrostatic screening of ions in solution, particularly divalent Mg, and can include complex contributions from multiple forces [46]. In simple terms, this electric double layer creates repulsive electrostatic interactions between the origami and surface. Increased electrostatic screening from increasing cation concentrations allows the repulsive barrier to be overcome, and structures become bound through van der Waals interactions. However, at our Mg concentrations, the DNA origami themselves exhibit minimal aggregation; but for Mg concentrations over ~20 mM at pH 6.6, the electrostatic repulsion between the DNA origami and the boron-implanted silicon surface is sufficiently reduced to allow adsorption. In contrast, the electric double layer of the thermal oxide, and thus, its repulsive electric potential barrier, is strongly dependent on the pH-dependent surface concentration of silanol-groups and adsorption of hydrated Mg ions [44]. Based on the work of Ong et al. [47], we calculated that ~20% of the silanol-groups at the surface would be deprotonated between pH values of 5.8 to 7.2. In comparison, at a pH value of 8.3, ~50% of the silanol-groups were deprotonated, giving rise to a more negative surface charge (see Supplement S10 for the derivation), increasing adsorption of hydrated Mg ions, and reducing the barrier for DNA origami adsorption. This is consistent with the pH-dependent deposition results, which are summarized in Figure 5.

## 4. Materials and Methods

### 4.1. DNA Origami Synthesis

The DNA origami triangle design was adopted/adapted from a previously reported study [24]. The design was modified to include 6 fluorescent 5’ FAM dyes (Integrated DNA Technologies, Skokie, IL, USA) to facilitate sample purification. DNA origami triangles were self-assembled from single-stranded scaffolds (Bayou Biolabs, Metairie, LA, USA), sourced from the M13mp18 bacteriophage, and staple strands (Integrated DNA Technologies, Skokie, IL, USA). The DNA scaffolds and corresponding staples were mixed in a 1:10 molar ratio, in a 1× tris-acetate-ethylenediaminetetraacetic acid (EDTA) buffer (Fisher Scientific, Hampton, NH, USA), with a pH of 8.3, and a [MgCl_2_] of 12.5 mM. The mixture was annealed at 70 °C for 20 min and then cooled to 20 °C at a rate of 0.6 °C/min. Well-formed nanostructures were purified using rate-zonal centrifugation as described in Supplement S1 [48,49]. After purification, the solution was normalized to a 5 nM concentration and stored in 5 µL aliquots at −80 °C to minimize sample degradation.

### 4.2. Substrate Cleaning

Silicon substrates were sonicated in deionized water, followed by 100% acetone (KMG, Fort Worth, TX, USA), and then 100% isopropanol (KMG, Fort Worth, TX, USA). The substrates were then sequentially soaked in Piranha to remove the organic contaminants and then 1:100 HF (Fisher Scientific, Hampton, NH, USA), to remove the surface oxide. For additional details, see Supplement S1.

### 4.3. DNA Origami Deposition

First, 20 µL of a deposition buffer was added to a 5 µL DNA origami solution with a 5 nM concentration. Once combined, the solution was gently pipette mixed. Cleaned silicon substrates were individually placed inside a petri dish (Fisher Scientific, Hampton, NH, USA) on top of a general-purpose lab wipe that was soaked in a deposition buffer so as to combat evaporation and subsequent change in the concentration of the deposited DNA solution. Then 25 µL of the DNA origami mixture was deposited onto the silicon substrates within 40 min of their cleaning, but before the native oxide grew back (Supplement S11). After deposition, the petri dish was sealed to minimize evaporation during incubation and sample transport between labs. For the MgCl_2_ concentration screening experiments, boron-implanted silicon samples were incubated at room temperature for ~24 h. For the pH screening experiments and the DNA origami deposition onto patterned substrates with boron-implanted silicon features, samples were incubated at room temperature for ~1 h. The DNA origami surface density on Piranha + HF cleaned boron-implanted silicon substrates, as a function of incubation time, is shown in Supplement S6. After incubation, excess DNA origami was removed by gravity-assisted rinsing, see Supplement S1.

### 4.4. Determining Surface Density

With the exception of boron-implanted silicon features surrounded by a raised SiO_2_ matrix, three high-resolution 5 µm × 5 µm images were captured in fluid AFM (Dimension FastScan, Bruker, Santa Barbara, CA, USA). Each image was divided into twenty-five 1 µm × 1 µm squares, and the number of DNA origami triangles in each square was manually counted. The average number of DNA nanostructures per 1 µm × 1 µm square is indicated by the average surface density.

## 5. Conclusions

Physical adsorption of DNA nanostructures was demonstrated on boron-implanted silicon substrates. While the average surface density was statistically comparable to natural mica, the adsorption uniformity was greater. The surface density dramatically increased from 0–91 nanostructures per µm^2^ as the salt concentration of the deposition buffer increased from 0–35 mM. Adsorption was also pH independent on the boron-implanted silicon surfaces, for the range we tested. The adsorption contrast between the boron-implanted silicon surface and SiO_2_ was optimized in a deposition buffer pH range of 5.8–7.2 at a [MgCl_2_] of 35 mM. In support of prior experiments, deprotonated silanol-groups at elevated pH in the presence of hydrated Mg ions promoted extensive binding between DNA origami and the oxide surfaces. Surface electrostatics provided by boron implantation enabled DNA adsorption, but further research is necessary to fully understand the electrochemical interactions controlling DNA origami deposition. Recommended next steps include further characterization of the boron-silicon surface structure, exploring how adsorption varies as a function of the feature size, and tuning the binding strength through mono and divalent cation concentrations to enable formation of ordered DNA origami arrays within the boron-implanted silicon wells [50,51,52].

## Supplementary Materials

Supplementary materials can be found at http://www.mdpi.com/1422-0067/19/9/2513/s1.

## Abbreviations

DNA	deoxyribonucleic acid
SiO_2_	silicon dioxide
MgCl_2_	magnesium chloride
[MgCl_2_]	magnesium chloride concentration
SADP	self-aligned double patterning
SAQP	self-aligned quadruple patterning
DSA	directed self-assembly
BCP	block copolymers
DSA-BCP	block copolymer directed self-assembly
APTES	aminopropyltrimethoxysilane
HMDS	hexamethyldisilazane
SAM	self-assembled monolayer
XPS	X-ray Photoelectron Spectroscopy
SIMS	Secondary Ion Mass Spectroscopy
TEM	Transmission Electron Microscopy
FIB	focused-ion beam
HF	hydrofluoric acid
FFT	Fast Fourier Transform
HCl	hydrochloric acid
AFM	atomic force microscopy
EDTA	ethylenediaminetetraacetic acid
FAM	fluorescein amidite
