# Supplementary material for: Boron-Implanted Silicon Substrates for Physical Adsorption of DNA Origami
Source: Int J Mol Sci. 2018 Aug 24;19(9):2513. doi: 10.3390/ijms19092513 (PMC6165417; doi:10.3390/ijms19092513)
Supplement: Supplementary file 1 [file ijms-19-02513-s001.pdf]

# Boron-Implanted Silicon Substrates for Physical Adsorption of DNA Origami

*Sadao Takabayashi[a], Shohei Kotani[a], Juan Flores-Estrada[a,b], Elijah Spears[a,b],*
*Jennifer E. Padilla[a],Lizandra C. Godwin[a], Elton Graugnard[a],*
*Wan Kuang[c], Scott Sills[b], William L. Hughes[a*]*

[a] Micron School of Materials Science & Engineering,
Boise State University, Boise, ID 83725, USA.

[b] Micron Technology, Inc., PO Box 6, 8000 South Federal Way,
Boise, ID 83707-0006, USA.

[c] Department of Electrical & Computer Engineering,
Boise State University, Boise, ID 83725, USA.

EMAIL: willhughes@boisestate.edu

**Supplement Table of Contents**

S1. Standard synthesis, purification, storage, cleaning, deposition, and sample rinsing procedures
S2. Fabrication of boron-implanted silicon, silicon dioxide, and patterned substrates
S3. XPS analysis of a boron-implanted silicon substrate
S4. Effect of boron-implantation on hydrophobicity of the substrate
S5. DNA origami adsorption on to mica vs. boron-implanted silicon
S6. DNA origami adsorption as a function of incubation time
S7. Standard nanostructure counting procedure of AFM images
S8. Figure for the pH-screening test with a full range of pH values tested
S9. Effect of oxide thickness on DNA origami triangle adsorption
S10. Correlation between deprotonated silanol-groups to the DNA origami adsorption
S11. Oxide growth analysis on boron-implanted silicon substrates
S12. Supplemental references

## S1. Standard synthesis, purification, storage, cleaning, deposition, and sample rinsing procedures

**DNA origami triangle synthesis -** The design of the DNA origami triangle was adapted from a previously reported study.[1] The design was slightly modified to include 6 fluorescent 5' FAM dyes to facilitate sample purification. The DNA origami triangles were self-assembled from a single-stranded scaffold (Bayou Biolabs), sourced from M13mp18 bacteriophage, and 208 unique staple strands (Integrated DNA Technologies). The scaffold and staples were mixed in 1:10 molar ratio, in 1x TAE (40 mM tris, 20 mM acetic acid, 2 mM ethylenediaminetetraacetic acid (EDTA); pH8.3), with a $MgCl_2$ concentration of 12.5 mM. After they were thoroughly mixed, the mixture was annealed at 70 ˚C for 20 min and cooled to 20 ˚C at a cooling rate of 0.6 ˚C/min. After annealing, the DNA origami was purified by rate-zonal centrifugation.[2]

**Rate-zonal centrifugation** – Eight layers of glycerol solution, in 1x TAE buffer with $MgCl_2$ concentration of 12.5 mM $MgCl_2$, were carefully laid into a 1.0 mL PC Thick-Walled tube (Thermo Scientific) starting with 60 vol.% solution at the bottom of the tube and 5 vol.% decrement per layer above. The layers were incubated at 4 ˚C for a minimum of 8 hours to turn them into a continuous gradient. A 160 µL of unpurified-DNA origami triangle solution was mixed with a 40 µL of 60 vol.% glycerol solution. After the mixture was thoroughly pipette mixed, it was gently dispensed on top of the gradient. The tube was then spun at 300,000 rcf for 45 min at 4 ˚C using Sorvall™ MTX 150 Micro-Ultracentrifuge (Thermo Scientific) with S140-AT fixed angle rotor (Thermo Scientific). After spinning, the unpurified DNA origami was separated into a thick band of excess DNA staple strands at the top of the gradient, a thin band of well-formed DNA origami triangles at the middle of the gradient, and a pellet of aggregated DNA origami triangles and DNA staples at the bottom of the tube. To visualize the bands of well-formed DNA origami triangles, the FAM dyes on the DNA origami were excited using a high intensity blue LED lamp. The excitation light from the LED lamp and the emission light from the sample were filtered using a 315 - 445 nm / 715 nm - 1095 nm band pass color filter (THORLABS) and a 550 nm long pass color filter (THORLABS), respectively, to help visualizing the fluorescence. FAM dyes were used instead of intercalating dyes to aid visualizing the DNA origami triangles because intercalating dyes are known to deform DNA origami. After the layer of excess staples was removed from the top of the gradient, the band of DNA origami triangles was extracted using a manual pipette.

The buffer of the extracted well-formed DNA origami solution was exchanged with 10 mM bis-tris HCl (BTH) buffer with the pH of 6.5, and a $MgCl_2$ concentration of 12.5 mM. For the buffer exchange, approximately 200 µL of well-formed DNA origami solution was placed in a 100 kDa Amicon Filter (EMD Millipore), and ~300 µL of BTH buffer was added to the filter. After the mixture was thoroughly pipette mixed in the filter, it was spun at 4,900 rcf for 3 minutes using Centrifuge 5430 R (Eppenforf). After the spin, the filtrate was discarded and the additional BTH buffer was added into the filter to fill it up. The buffer exchange cycle was repeated two more times to completely replace the buffer into the BTH. The purified DNA origami triangle was then extracted from the filter using a manual pipette.

**DNA origami storage** – The purified DNA origami triangles were normalized to 5 nM concentration using BioPhotometer (Eppendorf). The DNA origami triangle solution was then divided into 5 µL aliquots in 0.2 mL PCR tubes (Fisher Scientific), and then they were stored at -80 ˚C. This technique enabled us to perform a number of experiments using the same batch of DNA origami triangles to promote consistency in the experiments.

**Piranha + HF cleaning of the substrates** – A maximum of nine pieces of approximately 1 cm x 1 cm substrates were simultaneously cleaned by loading them into a custom-made Teflon substrate holder. First, the substrates were sequentially sonicated in DI water, 99.5% acetone cleanroom LP (KMG), and 100% isopropanol cleanroom LP (KMG) for 5 minutes using a table top sonicator. After the substrates

were rinsed in a DI wafer bath for 30 seconds, they were dipped into a Piranha solution, a mixture of 280 mL of 96% sulfuric acid (KMG) and 70 mL of 30% hydrogen peroxide (KMG), at approximately 90 ˚C for 10 minutes while agitating the solution with a magnetic stirrer. After the Piranha dip, the substrates were thoroughly rinsed in a DI bath for 5 minutes followed by a DI shower for 30 seconds. The substrates were then dipped into 1:100 hydrofluoric acid (HF) solution, a mixture of 150 mL of DI water and 150 mL of 1:50 HF solution (J.T Baker), for 2 minutes. The substrates were then rinsed in a DI bath followed by a through rinse in a DI shower for 30 seconds each. After cleaning, the substrates were dried with compressed air in preparation for DNA origami deposition.

**Piranha cleaning of the substrates** – A maximum of nine pieces of approximately 1 cm x 1 cm silicon substrates were simultaneously cleaned by loading them into a custom-made Teflon substrate holder. First, the substrates were sequentially sonicated in DI water, acetone, and isopropanol for 5 minutes each using a tabletop sonicator. After the substrates were rinsed in a DI wafer bath for 30 seconds, they were dipped into a Piranha solution at approximately 90 ˚C for 10 minutes while agitating the solution with a magnetic stirrer. After the Piranha dip, the substrates were thoroughly rinsed in a DI bath for 5 minutes followed by a DI shower for 30 seconds. The substrates were then dried with compressed air in preparation for the DNA origami deposition.

**DNA origami deposition** – Prior to the deposition, the aliquots of DNA origami triangles in the 0.2 mL PCR tubes were taken out of a -80 ˚C freezer and incubated at room temperature to thaw the solution. Right before the deposition, a 20 µL of deposition buffer was added to the PCR tube containing the 5 µL of DNA origami solution with a 5 nM concentration. Once combined, the solution was gently pipette mixed. Cleaned silicon substrates were individually placed in a petri dish (Fisher Scientific) on top of a general-purpose lab wipe (Fisher Scientific) that was soaked in a deposition buffer. Then 25 µL of the DNA origami mixture was deposited onto silicon substrates within 40 minutes of their cleaning (see above), before the oxide was observed to grow back. After deposition, the petri dish was sealed closed to minimize evaporation during incubation and sample transport between labs. For the $MgCl_2$ concentration screening experiments, boron-implanted silicon samples were incubated at room temperature for ~24 hours. For the pH screening experiments, all silicon samples were incubated at room temperature for ~1 hour. After incubation, excess DNA origami was removed by gravity-assisted rinsing.

**Gravity assisted rinsing** – For consistent rinsing, a fast-release pipette pump (Fisher Scientific) with a 10 mL disposable serological pipette (Fisher Scientific) was vertically fixed onto an apparatus (Figure S1). The height of the apparatus was adjusted, so the distance between the pipette tip and the silicon sample were ~1 cm. A 5 mL of rinsing buffer was aspirated into the pipette – the rinsing buffer was the same buffer as the deposition buffer for all samples. A small glass container (Fisher Scientific) was placed upside down on a 20 mL glass petri dish (Fisher Scientific) as a sample stage. The sample was placed on the top of the glass container, and it was placed directly underneath the pump. The air valve on the pump was then opened to release the rinsing buffer onto the sample. The waste rinsing buffer was collected in the petri dish below the stage, so the sample does not get contaminated from the waste

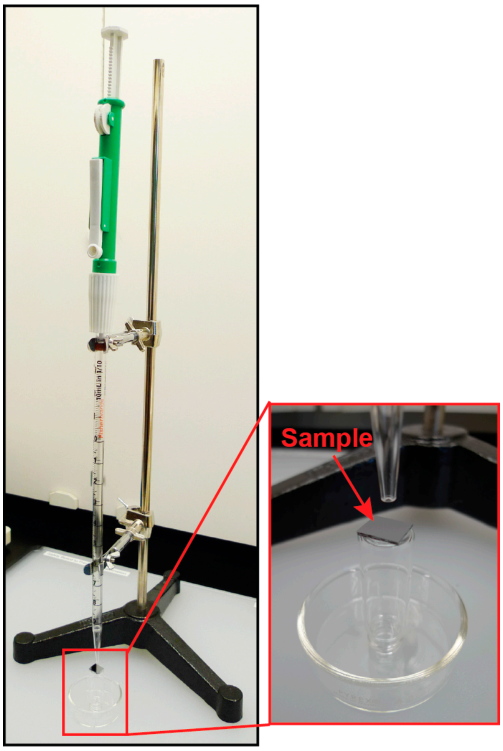


**Figure S1**. Two photographs of the gravity assisted rinsing apparatus. A consistent volume of rinsing buffer was aspirated by a fast-release pipette pump and dispensed onto the silicon sample by opening the air valve. The silicon substrate was placed onto the bottom of a glass container that was placed upside down on a glass petri dish. For all silicon substrates, the volume of rinsing buffer was 5 mL, and the distance from the pipette tip to the sample was fixed to ~1 cm.

## S2. Fabrication of boron-implanted silicon, silicon dioxide, and patterned substrates

The boron-implanted silicon substrate, $SiO_2$ substrate, and the substrate with boron-implanted silicon features surrounded by a $SiO_2$ matrix were prepared at Micron Technology from 300 mm p-type silicon wafers with $1x10^6$ boron per $cm^3$. Listed below is a brief description of each wafer.

(1) For the boron-implanted silicon substrate, $1x10^{17}$ boron ions per $cm^2$ were implanted into a p-type wafer with an energy of 500 eV. The implanted wafer was then annealed at 900˚C for 20 seconds in a 95% nitrogen environment that contained 5% oxygen in support of ion damage recovery.

(2) The $SiO_2$ substrate was prepared by annealing a p-type wafer at 900˚C to fabricate a 100 nm thick $SiO_2$ layer at the surface of the substrate. The $SiO_2$ layer was thermally grown rather than deposited onto the wafers to achieve sub-nanometer surface roughness.

(3) The substrate with boron-implanted silicon features surrounded by a $SiO_2$ matrix was fabricated by first preparing a thermally grown $SiO_2$ wafer with the process described in (2). A 480 nm thick layer of negative photoresist was then spun onto the wafer. Patterns on a reticle mask were transferred onto the photoresist via photolithography. After developing the photoresist, the $SiO_2$ on the exposed region was completely removed using Inductively Coupled Plasma – Reactive Ion Etching (ICP-RIE). This step exposed the p-type silicon underneath the $SiO_2$. The boron ions were implanted into the exposed p-type silicon surface with a dose of $2x10^{16}$ boron ions per $cm^2$ with an energy of 500 eV. The fabrication process for the boron-implanted features surrounded by the $SiO_2$ matrix is shown in Figure S2. In addition, an optical image of the boron-implanted silicon features, surrounded by a raised $SiO_2$ matrix, is shown in Figure S3. The AFM images in Figure 4 were acquired from the highlighted features in Figure S3.

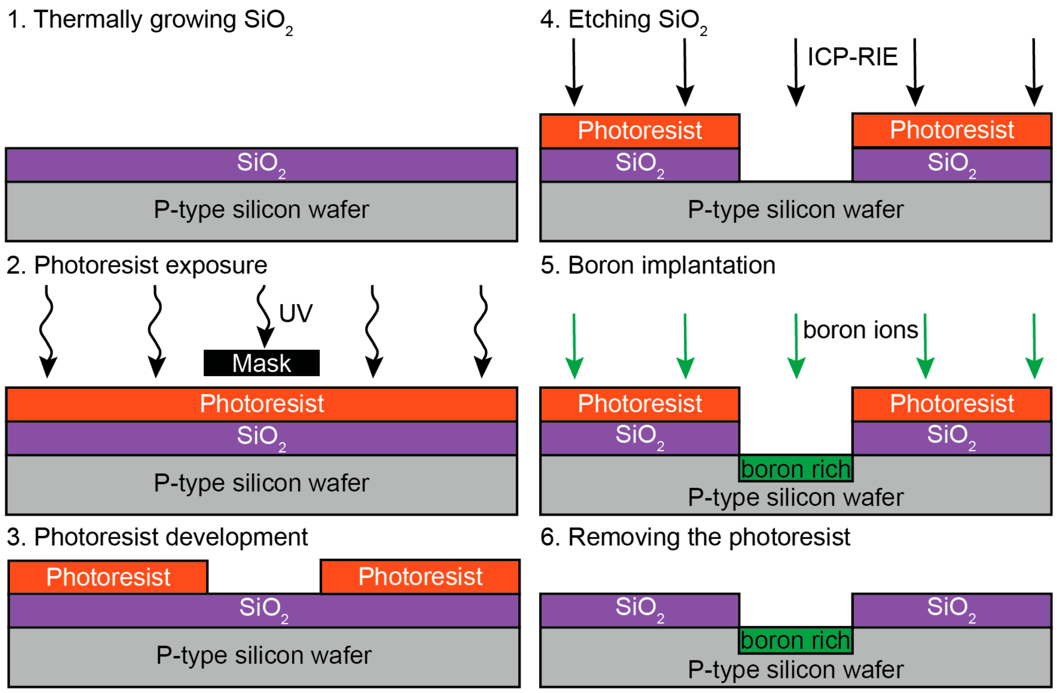


**Figure S2.** A schematic of the process flow used by Micron Technology to create the silicon substrate with boron-implanted features surrounded by a $SiO_2$ matrix. The features are not to scale.

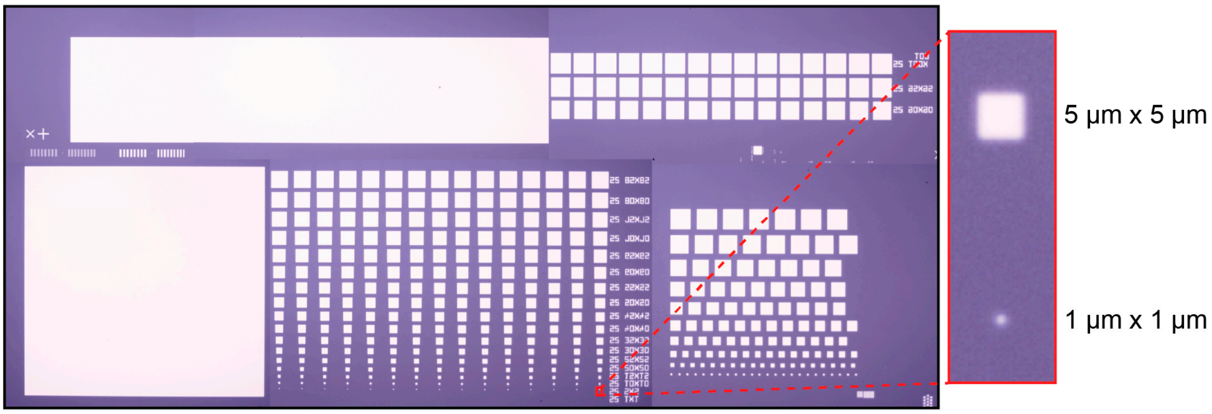


**Figure S3.** Optical image of boron-implanted silicon features surrounded by a raised $SiO_2$ matrix at low magnification. The square features where the AFM images were acquired in Figure 4 are highlighted in red. Stitching together six optical images generated the low magnification image.

## S3. XPS analysis of a boron-implanted silicon substrate

A boron-implanted silicon substrate was analyzed using X-ray Photoelectron Spectroscopy (XPS) to characterize the surface. The characterization of the boron-implanted silicon substrate was performed at Micron Technology. The XPS analysis of the as-received boron-implanted silicon substrate showed that the oxide thickness was ~2.6 nm (data not shown). The XPS analysis of the substrates, after removing the boron-rich oxide, revealed that the boron concentration was ~2.5 atomic percent at the surface, and it was statistically equivalent across all three substrates that were analyzed (Table S1). The XPS survey spectrums of all three substrates are shown in Figure S4. The detected surface contaminations were intentionally not included in the boron concentration calculation. The 3D boron concentration map of the substrate, generated using SIMS, also revealed a uniform distribution of boron across 500 µm x 500 µm area of the surface without the boron-rich amorphous oxide layer (Figure S5). Based on the analysis, the boron concentration distribution the substrate surface was uniform.

**Table S1**. Surface boron concentration of three, ~1 cm x 1 cm substrates after removing the amorphous oxide using HF

| Substrate | Data Points | Average Boron Concentration (atomic%) |
|---|---|---|
| A | 5 | 2.3 ± 0.2 |
| B | 5 | 2.6 ± 0.2 |
| C | 5 | 2.7 ± 0.2 |

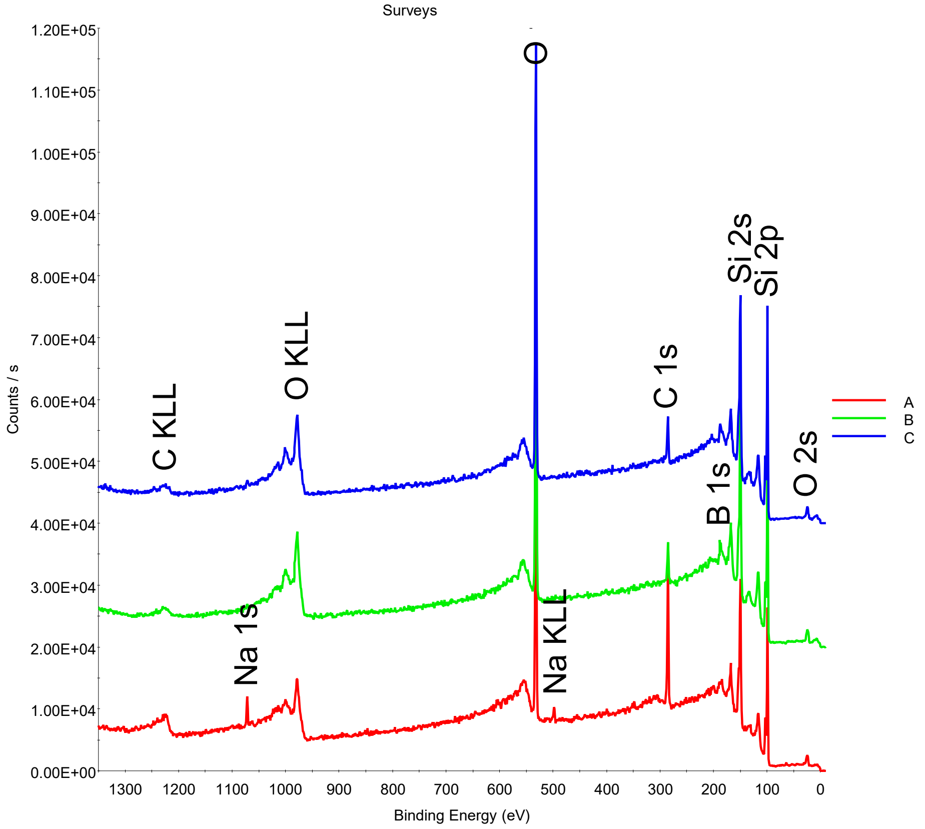


**Figure S4.** The XPS spectra from the three substrates shown in Table S1.

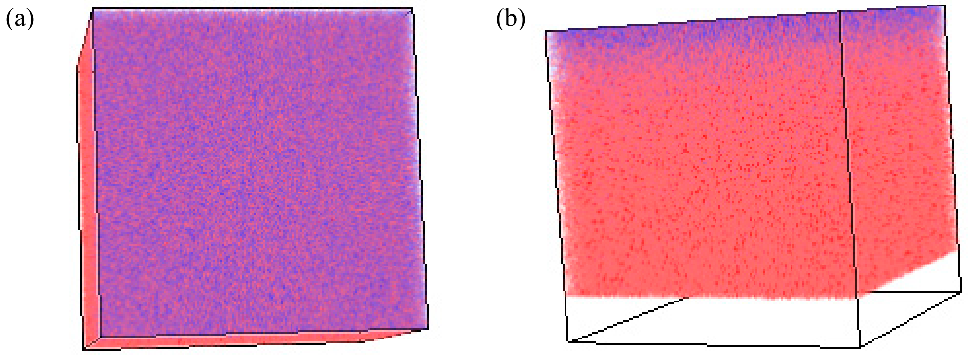

**Figure S5.** (a) The top view and (b) the side view of the 3D boron concentration map of boron implanted silicon substrate after removing the naturally-grown oxide, generated using SIMS. The boron is shown in blue and silicon is shown in red. The map clearly shows a uniform distribution of boron on the substrate surface. The analyzed area was 500 µm x 500 µm.

## S4. Effect of boron-implantation on hydrophobicity of the substrate

A droplet of deionized water was deposited onto a substrate before boron-implantation (p-type silicon) and a substrate after boron-implantation (boron-implanted silicon), shown in Figure S6. The contact angle of the p-type silicon substrate was greatly reduced by boron-implantation. The boron-rich layer at the surface induces a surface potential, thus the surface become hydrophilic after boron-implantation.[3]

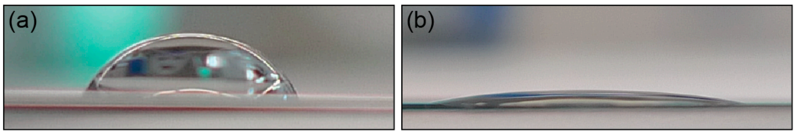


**Figure S6**. Optical images of a deionized water droplet deposited onto (a) a p-type boron-doped ($10^{16}$ /cm3) silicon substrate and (b) a boron-implanted silicon substrate. In (a) and (b), the contact angle of p-type silicon was far greater than the contact angle of boron-implanted silicon substrates. The boron-implantation process reduced the hydrophobicity of the substrate. The substrates were first cleaned by a standard procedure described in Supplement S1 before depositing deionized water.

## S5. DNA origami adsorption on to mica vs. boron-implanted silicon

DNA origami was deposited onto freshly cleaved mica and Piranha + HF cleaned boron implanted silicon to compare the average surface density. While the average DNA origami surface density of boron implanted silicon was comparable to that of mica, the adsorption uniformity was greater, as shown in Figure S7. For both samples, 10 mM BTH buffer with pH value of 6.6 and $MgCl_2$ concentration of 35 mM was used as a deposition buffer, and they were incubated at room temperature for ~24 hours.

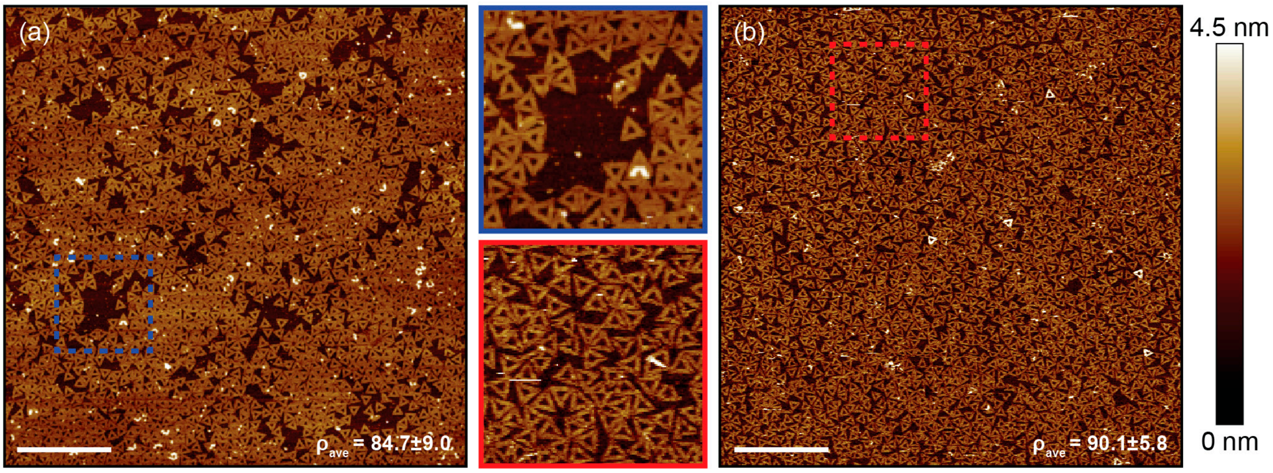


**Figure S7.** AFM images and insets of DNA origami triangles that adsorbed onto: (a) freshly-cleaved mica, and (b) Piranha + HF cleaned boron-implanted silicon substrates. For both surfaces, the deposition buffer was 10 mM BTH buffer with pH value of 6.6 and $MgCl_2$ concentration of 35 mM. The deposition incubation time was ~24 hours. The average surface density on the boron-implanted silicon was 90 ± 6 / $\mu m^2$ and was statistically equivalent to that of the mica. Scale bars are 2 μm and the insets are 2 μm x 2 μm.

## S6. DNA origami adsorption as a function of incubation time

The optimum incubation time was determined by depositing DNA origami triangles onto boron-implanted silicon substrates when the incubation time varied from 1 minute to 7 days. A plot of surface density as a function of incubation time and corresponding AFM images are shown in Figure S8. The cleaning of the boron-implanted silicon substrates and the DNA origami deposition was performed by the standard procedures, described in Supplement S1. The deposition buffer and the rinsing buffer was 10 mM BTH buffer with pH value of 6.6 and $MgCl_2$ concentration of 35 mM. After incubation, the sample was rinsed with the rinsing buffer using the gravity assisted apparatus.

A rapid increase of the surface density was observed until 25 hours of incubation when it reached a maximum surface density of ~90 structures per $\mu m^2$. After 25 hours, the surface density slowly decreased until 4 days of incubation, where the system was assumed to reach its equilibrium state. The surface density at the assumed equilibrium state was ~70 structures per $\mu m^2$. The results from our oxide growth analysis in Supplement S11 indicate that there was no growth in the oxide for a week of incubation when the substrate surface was covered with buffer containing $MgCl_2$. Therefore, the oxidation of the substrate surface was not the reason for the reduction in the DNA origami adsorption between 25 hours and 4 days of incubation. No further study was done to identify the cause for this reduction.

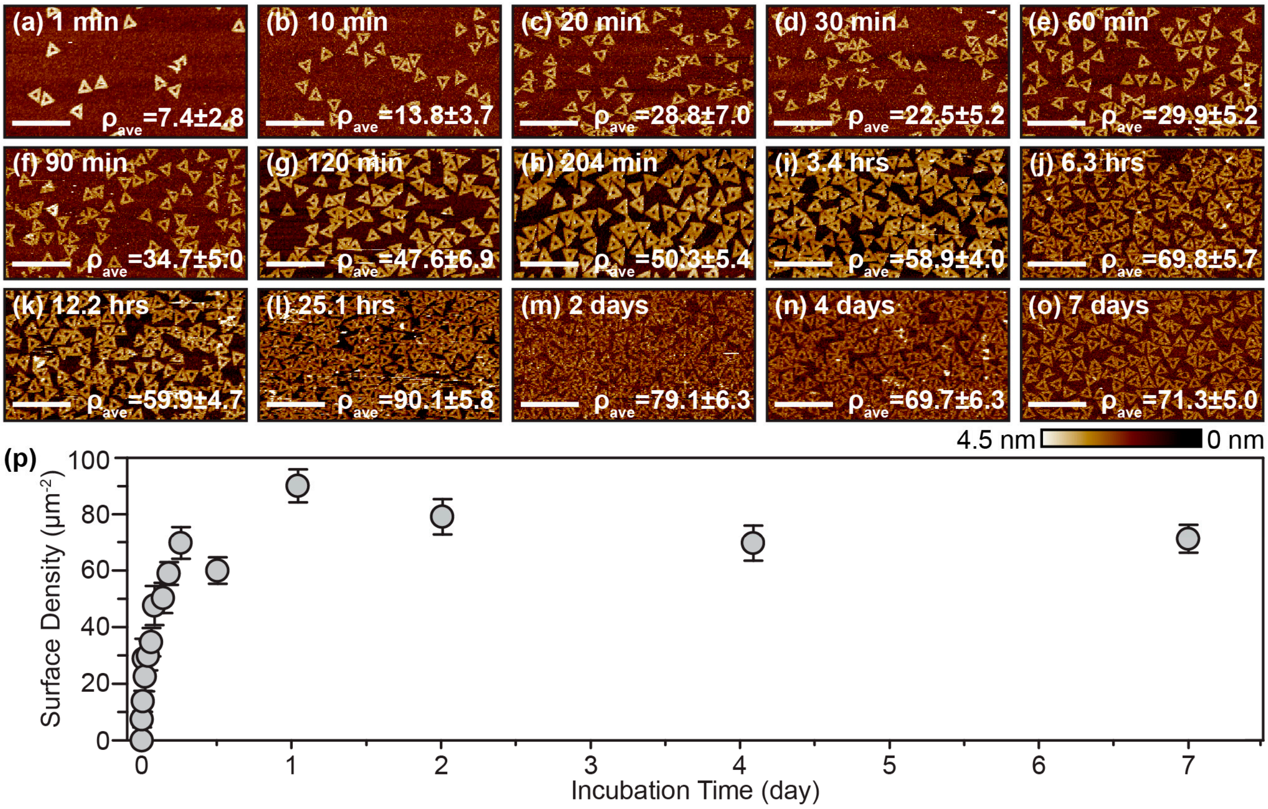


**Figure S8**. AFM images of DNA origami nanostructures deposited onto boron-implanted silicon substrates with incubation times varying from 1 minute to 7 days (a - o), and a plot of the surface density as a function of the incubation time (p). The DNA origami adsorption increased rapidly in the first 25 hours of incubation, where the maximum surface density was observed. The surface density was gradually reduced from 25 hours of incubation to 4 days, where the system appeared to reach its equilibrium state. Scale bars are 500 nm.

## S7. Standard nanostructure counting procedure of AFM images

Three 5 µm x 5 µm high-resolution AFM images were acquired in fluid from each sample. The images were digitally modified using Nanoscope Analysis software (Bruker) to maximize the contrast between the DNA origami triangles and the background. The resulting color AFM images were converted to grayscale images using Photoshop (Adobe) and then imported into Illustrator (Adobe) to superimpose a 1 µm x 1 µm grid onto them. The grid divided the AFM images into 25 – 1 µm x 1 µm sections for ease of counting. The AFM images were then opened in ImageJ (National Institutes of Health) to manually count the number of DNA origami triangles in each section using the cell counter plugin. During counting, a marker was placed onto each DNA nanostructure to prevent redundant counting, see Figure S9. The result was summarized in Excel (Microsoft) to determine the surface density ($\rho_{ave}$) - average number of DNA origami triangles in each 1 µm x 1 µm section.

Counting rules were created to minimize human factors across the counters as listed below.

- Ignore aggregated DNA origami when individual nanostructures cannot be distinguished.
- Count partially missing DNA nanostructures when nanostructure drift was observed during AFM imaging.

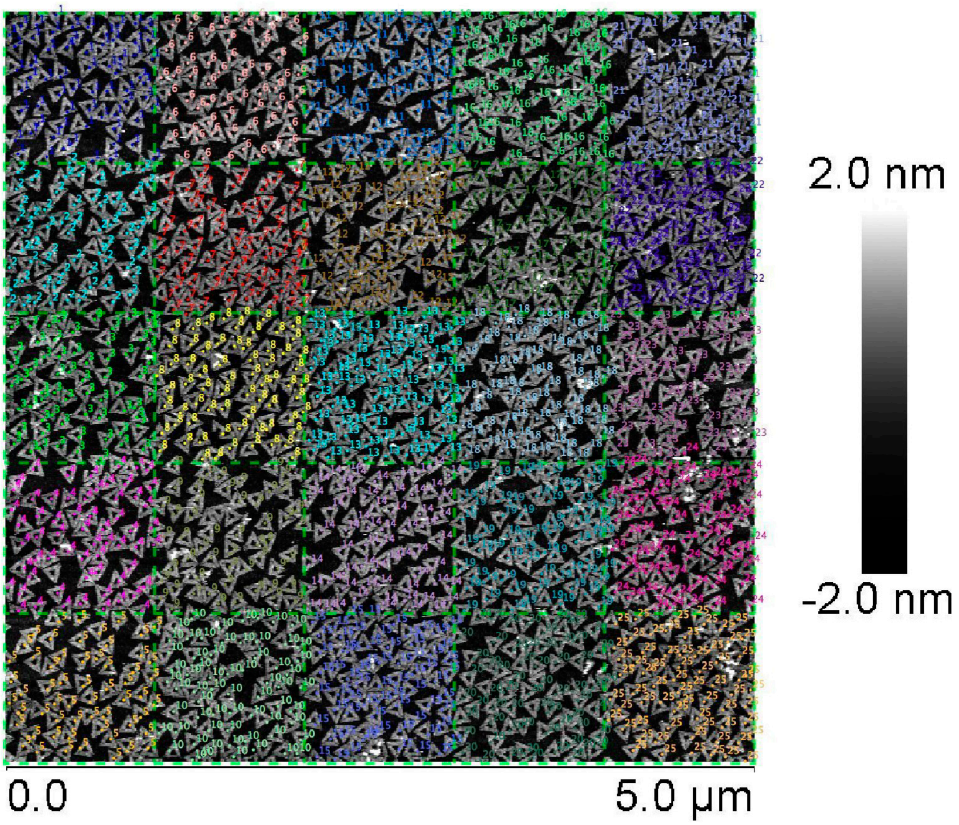


**Figure S9.** Example of a processed AFM image used for counting the DNA nanostructures. The 5 µm x 5 µm AFM height image was divided into 25 – 1 µm x 1 µm sections using a grid shown in green dotted lines. A marker was placed onto each DNA origami triangle after it was counted to prevent double counting.

## S8. Figure for the pH-screening test with a full range of pH values tested

A figure for the pH-screening test with the full range of pH value tested is shown in Figure S10.

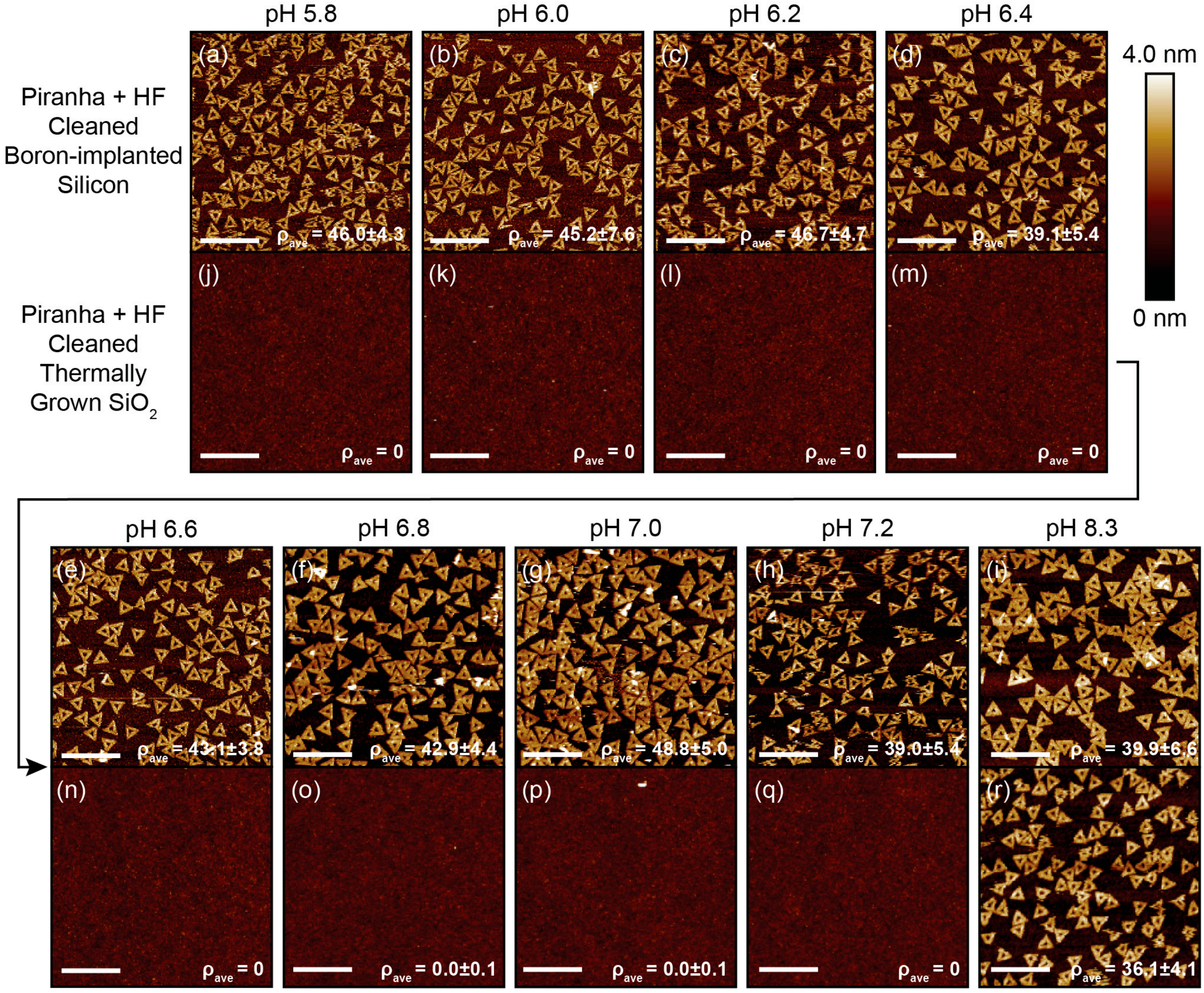


**Figure S10**. AFM images of DNA origami triangles that were deposited onto the boron-implanted silicon substrates (a - i) and the $SiO_2$ substrates (s - aa). The pH of the deposition buffers varied between 5.8 and 8.3. The DNA origami triangles consistently adsorbed onto the boron-implanted silicon substrates. In contrast, the DNA origami adsorption on the $SiO_2$ substrates were minimum between the pH of 5.8 and 7.2. The surface densities were reported by measuring the average number of structures per unit area ($\rho_{ave}$) from three high-resolution 5 µm x 5 µm AFM images. Scale bars are 500 nm.

## S9. Effect of oxide thickness on DNA origami triangle adsorption

The oxide thicknesses were measured on the as-received boron-implanted silicon substrates that were cleaned with 1:100 HF from 30 seconds up to 4 minutes. The optimum HF cleaning time for removing the amorphous oxide layer on the substrate was determined. The boron-implanted silicon substrates were first cleaned with Piranha using the standard protocol, described in Supplement S1. The substrates were then cleaned with 1:100 HF for 30 seconds, 1 minute, 2 minutes, and 4 minutes. After the substrates were thoroughly rinsed and dried with nitrogen gas, the thicknesses of the remaining oxide layer were estimated using a spectroscopic ellipsometry (M-2000, J.A. Woollam). The spectra were fitted with a structure model that consisted of three layers: (1) a $SiO_2$ layer, (2) an intermediate $SiO_2$ layer, and (3) a 1000 µm thick silicon substrate. While the silicon substrate thickness was fixed, the thicknesses of both the $SiO_2$ and the intermediate layers were determined by the fitting process.

The oxide thickness exponentially decreased up to 2 minutes by dipping in a HF solution. Then the decrease in the oxide thickness plateaued beyond 2 minutes of dipping, Figure S12a. Therefore, the oxide on the surface of the boron-implanted silicon substrate was assumed to be completely removed after 2 minutes of HF cleaning.

The DNA origami triangles were deposited onto each substrate to determine the effect of amorphous oxide on DNA origami adsorption. The DNA origami was deposed onto the substrate using the standard procedure, described in S4. After the samples were incubated at room temperature for 1 hour, they were rinsed using gravity assisted rinsing. The DNA origami did not adsorb on the boron-implanted silicon substrates when they were cleaned with HF solution for less than 30 seconds. The DNA origami began to absorb onto the surface after 1 minute of HF cleaning, and it reached the maximum surface density of ~43 nanostructures per $\mu m^2$ at 2 minutes of HF dipping, Figure S12e.

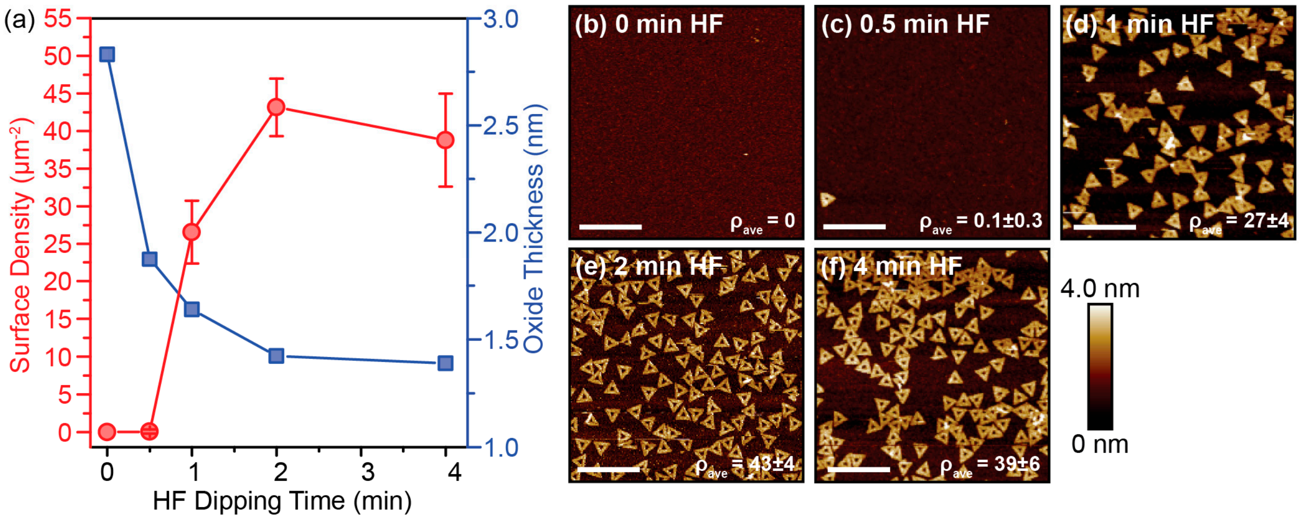


**Figure S12.** A plot of estimated amorphous oxide thickness on boron-implanted silicon substrates (a, blue line) and the DNA origami surface density on the boron-implanted silicon substrates (a, red line) as a function of HF dip time. The color of the lines indicates the corresponding vertical axis. The AFM images of DNA origami triangles deposited onto the corresponding substrates (b-f) are also shown. The oxide thickness decreased exponentially until 2 minutes of HF dipping – after which the oxide was assumed to be removed. The DNA origami surface density rapidly increased from 1 minute of dipping in HF and reached a maximum surface density at 2 minutes of dipping in HF. Scale bars are 500 nm.

## S10. Correlation between deprotonated silanol-groups to the DNA origami adsorption

According to Ong *et al.*, 19% of the silanol-groups on a $SiO_2$ surface possess a $pK_a$ of 4.5 and 81% of silanol-groups possess a $pK_a$ of 8.5.[4] For convenience, the silanol-groups possessing $pK_a$ of 4.5 are expressed as silanol-group 1, and the others are expressed as silanol-group 2 in this study. Assuming the properties of silanol-groups on our substrates are the same as Ong *et al.*, the overall population fraction of the deprotonated silanol-groups on a $SiO_2$ substrate surface is given by:

$$F_D = x_1 \times f_{D1} + x_2 \times f_{D2} \tag{S1}$$

where the $F_D$ is the overall population fraction of deprotonated silanol-groups on the entire substrate surface. The $x_1$ and $x_2$ are the population fraction of silanol-groups 1 and 2 on the entire substrate surface, respectively. Finally, the $f_{D1}$ and $f_{D2}$ are the fraction of the deprotonated silanol-groups within silanol-group 1 and silanol-group 2, respectively.

At equilibrium, the relationships between the pH of the buffer and the $pK_a$ of a silanol-group can be expressed by Henderson–Hasselbalch equation, given by:

$$\text{pH} = \text{p}K_{ai} + \log_{10}\left(\frac{[\text{A}_i^-]}{[\text{HA}_\text{i}]}\right) \tag{S2}$$

where the pH is the pH of the buffer. The $pK_{ai}$ is the $pK_a$ of silanol-group $i$. The $[A_i^-]$ and $[HA_i]$ are the concentrations of deprotonated and protonated silanol-group $i$, respectively.

By expanding Eqn. S1 and reorganizing Eqn. S2, the two equations become:

$$F_D = x_1 \frac{[\text{A}_1^-]}{[\text{HA}_1]+[\text{A}_1^-]} + x_2 \frac{[\text{A}_2^-]}{[\text{HA}_2]+[\text{A}_2^-]} \tag{S3}$$

$$\frac{[\text{A}_i^-]}{[\text{HA}_\text{i}]} = 10^{\text{pH}-pK_{ai}} \tag{S4}$$

By plugging in Eqn. S4 into Eqn. S3, the overall population fraction of deprotonated silanol-groups on the entire substrate surface is expressed as:

$$F_D = x_1\left(\frac{10^{\text{pH-pK}_{\text{a1}}}}{10^{\text{pH-pK}_{\text{a1}}}+1}\right) + \text{x}_2\left(\frac{10^{\text{pH-pK}_{\text{a2}}}}{10^{\text{pH-pK}_{\text{a2}}}+1}\right) \tag{S5}$$

As a silanol-group on the surface become deprotonated, it also becomes monovalent.[4] Therefore, the surface charge should be linearly proportional to the overall population fraction of the deprotonated silanol-groups, $F_D$.

The DNA origami triangles were deposited onto boron-implanted silicon substrates and $SiO_2$ substrates using deposition buffers with the pH ranging from 5.8 to 8.3. The substrates were cleaned by the standard procedures, and the samples were rinsed using the gravity assisted apparatus. For the DNA origami deposition with the deposition buffer pH between 5.8 and 7.2, 10 mM BTH buffers with a $MgCl_2$ concentration of 35 mM were used. For the DNA origami deposition with the deposition buffer pH of 8.3, 1x TAE buffer with a $MgCl_2$ concentration of 35 mM was used. We assumed that the type of deposition buffer does not affect the DNA origami adsorption onto the substrates.

Between the deposition buffer pH values of 5.8 to 7.2, only 20 - 23% of the silanol-groups on the surface were deprotonated, indicating that the surface charge from the deprotonated silanol-groups was weak. As expected, nearly zero DNA origami adsorption was observed on $SiO_2$ substrates between the pH of 5.8 and 7.2. When the deposition buffer pH of 8.3, ~51% of the silanol groups were deprotonated – the surface charge roughly doubled. As a result, the DNA origami adsorption on $SiO_2$ substrates increased to ~40 structures per $\mu m^2$ - reaching an equivalent DNA origami surface density as the boron-

implanted silicon substrate. Since the DNA origami triangle adsorption on the $SiO_2$ substrates closely resembles the increase in the surface charge from the deprotonation of the silanol-groups, the DNA origami adsorption on the substrate is expected to be controlled by the deprotonation of the silanol-groups on the substrates. Even the extremely thin oxide layer on the boron-implanted silicon substrate surface can drastically alter the DNA origami triangle adsorption, as also shown in Supplement S9.

## S11. Oxide growth analysis on boron-implanted silicon substrates

The oxidation of the boron implanted silicon substrate was analyzed in three different conditions. The first condition was the ambient lab environment that occurred between the substrate cleaning and the DNA origami deposition. The second condition was after the surface was covered with deionized (DI) water to measure the effects of being stored in DI water. The third condition was after the surface was covered with a $MgCl_2$ containing buffer to measure the incubation effects after the DNA origami solution was deposited. The optical spectra from the substrate surface were acquired using a M-2000 Ellipsometer (J.A. Woollam), and the optical spectra were analyzed using the built-in software. The same theoretical model described in Supplement S9 was used to determine the oxide thickness. Here, the $SiO_2$ layer thickness from the fit is shown in Figure S13ab, as the oxide thickness of the substrate. The mean square error of each measurement was ~3.2 $nm^2$. Because the structure model does not account for the high boron concentrations and does not perfectly describe the actual substrate structure, there was about 1.4 nm of oxide still observed after the HF cleaning. Although the result might not represent the actual oxide thickness on the substrate, we believe is closely reflects relative thickness changes of the oxide.

Before starting the experiments, the substrates were cleaned with Piranha and HF following the protocol described in Supplement S1. After cleaning, the substrates were thoroughly dried with filtered compressed air.

**Monitoring the oxide growth in ambient conditions** - The oxide thickness measurement started directly after the substrate was cleaned and dried. The oxide thickness was periodically measured for 20 hours.

**Monitoring the oxide growth for the substrate surface covered with water** – After the substrate was cleaned and dried, 40 µL of DI water was deposited onto the substrate to cover its surface. The substrate was then stored on a general-purpose lab wipe in a sealed petri-dish. The lab wipe was soaked with DI water to prevent evaporation of the DI water on the substrate. Before each measurement, the substrate was thoroughly dried with compressed nitrogen gas. After the optical spectrum was measured, 40 µL of DI water was re-deposited onto the substrate to minimize the exposure to the ambient environment. For this sample, the optical spectrum was periodically captured for one week. The substrate was exposed to ambient air for ~3 minutes per measurement, so the total exposure to ambient air accumulated as the number of measurements increased.

**Monitoring the oxide growth for the substrates covered with buffer** – Soon after the substrate was cleaned and dried, 40 µL of 10 mM BTH buffer with pH of 6.4 and 35 mM $MgCl_2$ was deposited onto the substrate to cover up the substrate. The substrate was stored on a general-purpose lab wipe in a sealed petri-dish. The lab wipe was soaked with the same buffer to prevent evaporation of the sample. Before each measurement, the substrate was rinsed with DI water to eliminate the buffer solution on the surface and thoroughly dried with compressed nitrogen gas. After measurement, 40 µL of the buffer was re-deposited onto the substrate surface to minimize the exposure of the substrate to the ambient environment. For this sample, the oxide thickness was also measured for one week. The substrate was exposed to the ambient air for ~3 minutes per measurement.

In case multiple drying and rehydration cycles affected the oxide growth, three control substrates were prepared for each wet condition. The control samples were prepared and stored in the same way as the experimental samples, but unlike experimental samples, only one oxide thickness measurement was made on each control samples. This eliminated the effect of drying and rehydration process.

The oxide thickness of the boron-implanted silicon sample incubated in the dry ambient condition shows that the growth of the oxide was prevented for the first 40 minutes after HF cleaning, Figure S13a. When the sample incubated with DI wafer on the surface, no indication of oxide growth was shown for at least 7 days of incubation – shown in the blue plot in Figure S13b. In addition, a consistent DNA origami adsorption was observed on substrates stored in DI water at least for up to 14 days. For the sample incubated with a buffer on the surface, the increase in the oxide thickness was observed 5 days after it was cleaned with the HF – shown in the red plot in Figure S13b. However, the oxide thickness of the control samples did not change even after 7 days - shown by the orange diamonds. Thus, the increase in the oxide thickness was most likely due to the repeated drying and rehydrating cycles of the experimental sample and not from the oxidation during the incubation. Therefore, no oxide growth was observed on the substrate surface covered with a buffer containing $MgCl_2$, at least for one week. The results indicate that no oxide growth should be expected during the incubation of the sample after the DNA origami triangles deposition onto the substrate using standard deposition procedure.

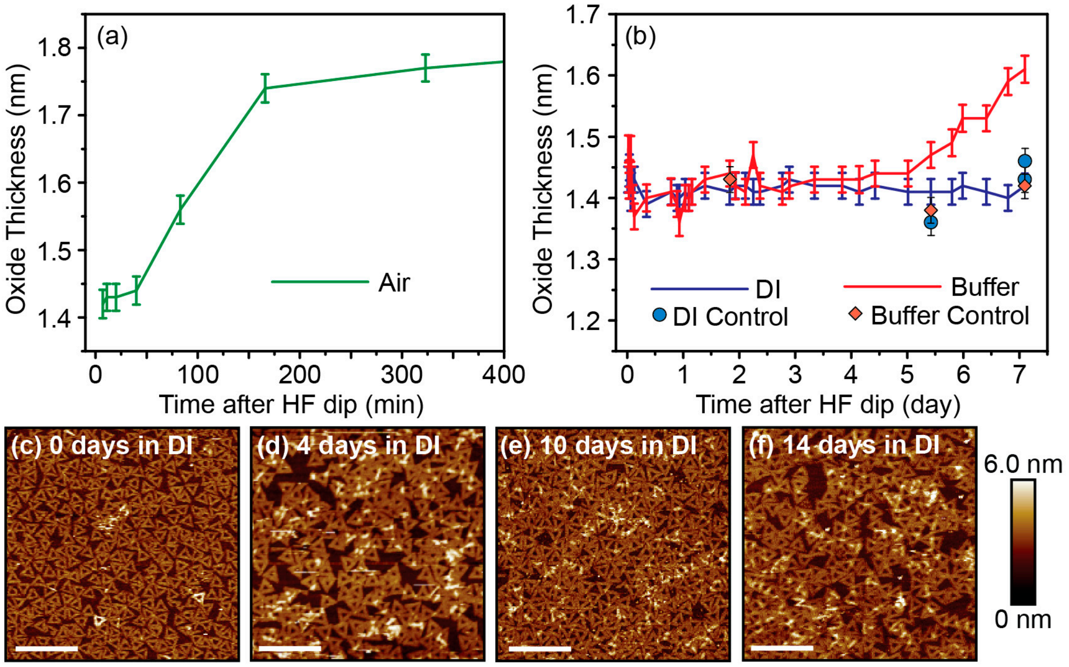


**Figure S13**. Oxide thickness on boron-implanted silicon substrates as a function of time after HF cleaning (a, b) and the AFM images of DNA origami adsorption on boron-implanted silicon substrates stored in DI water for up to two weeks (c – f). The substrates were incubated in an ambient air (a, green), incubated while the surface was covered with DI water (b, blue), and incubated while the surface was covered with a buffer containing $MgCl_2$ (b, red). The blue dots and the orange dots in (b) represent the data points from the control samples that were covered with DI water and buffer, respectively. A consistent DNA origami attachment was observed on boron-implanted silicon substrates stored in DI water at least for up to two weeks. Scale bars are 500 nm.

## S11. Supplemental references

1. Rothemund, P. W. K. Folding DNA to create nanoscale shapes and patterns. *Nature* **2006**, *440*, 297–302, doi:10.1038/nature04586.
2. Lin, C.; Perrault, S. D.; Kwak, M.; Graf, F.; Shih, W. M. Purification of DNA-origami nanostructures by rate-zonal centrifugation. *Nucleic Acids Res.* **2013**, *41*, 1–6, doi:10.1093/nar/gks1070.
3. Fujii, M.; Sugimoto, H.; Hasegawa, M.; Imakita, K. Silicon nanocrystals with high boron and phosphorus concentration hydrophilic shell—Raman scattering and X-ray photoelectron spectroscopic studies. *J. Appl. Phys.* **2014**, *115*, 084301, doi:10.1063/1.4866497.
4. Ong, S.; Zhao, X.; Eisenthal, K. B. Polarization of water molecules at a charged interface: second harmonic studies of the silica/water interface. *Chem. Phys. Lett.* **1992**, *191*, 327–335, doi:10.1016/0009-2614(92)85309-X.
